# Are There Clinical Differences in Limited Systemic Sclerosis according to Extension of Skin Involvement?

**DOI:** 10.1155/2014/716358

**Published:** 2014-11-11

**Authors:** Marina Scolnik, Luis J. Catoggio, Eliana Lancioni, Mirtha R. Sabelli, Carla M. Saucedo, Josefina Marin, Enrique R. Soriano

**Affiliations:** ^1^Rheumatology Section, Medical Services, Hospital Italiano de Buenos Aires, 1181 Buenos Aires, Argentina; ^2^Fundación Dr. Pedro M. Catoggio para el Progreso de la Reumatologia, 1181 Buenos Aires, Argentina; ^3^Instituto Universitario Escuela de Medicina, Hospital Italiano de Buenos Aires, 1181 Buenos Aires, Argentina

## Abstract

*Objectives*. To examine the characteristics of our patients with limited systemic sclerosis (lSSc) for differences between Barnett Type 1 (sclerodactyly only) and Type 2 or intermediate (acrosclerosis-distal but may reach up to elbows and/or knees plus face) subsets. *Methods*. Records of patients between January 1, 2000, and December 31, 2011, with SSc or those with anti-Scl-70, anticentromere, or antinucleolar antibodies were reviewed. Only cases fulfilling ACR 1980 criteria were included and classified as diffuse or limited according to LeRoy's criteria. Limited SSc was separated into sclerodactyly and acrosclerosis (Barnett's Types 1 and 2). *Results*. 234 SSc patients (216 females) fulfilled criteria. Female/male ratio was 12 : 1; 24% had dSSc and 76% lSSC (64% Type 1 and 12% Type 2). Total follow-up was 688 patient-years. Within lSSC, the Type 2 group had significantly shorter duration of Raynaud's and more anti-Scl-70 and less anticentromere antibodies. In particular, interstitial lung disease (ILD) was significantly more prevalent in Type 2 group and similar to Type 3. *Conclusions*. These results appear to confirm that extension of skin involvement within limited SSc may identify two different subsets with clinical and serologic characteristics.

## 1. Introduction

There appears to have been consensus in the past decades in classifying systemic sclerosis (SSc) according to extension of skin involvement as limited and diffuse, using the elbows and knees as “limits” to distinguish between them. However, although this has been used to “dissect” two more or less distinct clinical subsets, there are patients who do not fit this classification and several others have been proposed [1, 2].

The growing demonstration that autoantibodies specific for SSc such as anticentromere, anti-Scl-70 (anti-topoisomerase 1), and more recently a variety of antinucleolar antibodies, particularly anti-RNA polymerase 3, correlate with these subsets and/or visceral manifestations has led to them being used to “predict” clinical subsets in early disease and also raise awareness for the possibility of certain organ involvement (anti-Scl-70 and interstitial lung disease [3, 4], anticentromere associated with digital ulcers [5], debatably pulmonary hypertension [6], and anti-RNA polymerase 3 with renal crisis [7]).

**Figure 1:**
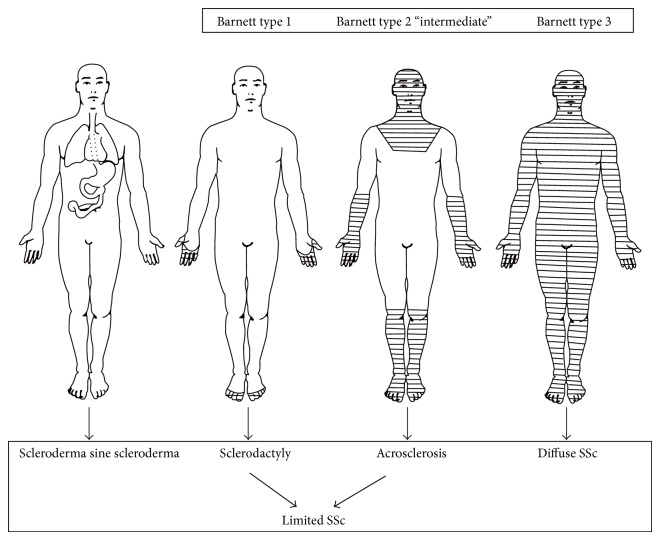
Clinical Subsets according to extension of skin involvement.

Decades ago, Barnett classified SSc as Type 1 (only sclerodactyly), Type 2 (acrosclerosis-distal but may reach up to elbows and/or knees plus face), and Type 3 (diffuse skin involvement) (Figure 1). Patients with Type 2 had an intermediate degree of both skin and organ involvement compared to Type 1 (less) and Type 3 (more) [8–10]. This classification by Barnett was extensively discussed in an editorial by Masi many years later [11].

The division into three categories according to extension of skin involvement was not agreed to by others, who could not find real differences between what we could call types 1 and 2 as determined by Barnett [1, 2]. A similar classification but this time with 4 categories, proposed by Giordano et al. [12], was also not supported for similar reasons. Simultaneously, as reasonable stress was laid on early disease detection, the extent of cutaneous involvement was perhaps not considered so relevant, particularly as opposed to the autoantibodies as subset identifiers [2].

However, truth is that many patients present with evolving disease, and all of SSc “specific” antibodies are not widely available in many parts of the world, the best example being anti-RNA polymerase. Thus, in practice, a clinical subsetting may still be useful.

Many years ago but with a small number of patients, we noted that the limited subgroup could be, indeed, separated clinically as Barnett had suggested according to skin involvement into patients with “only fingers,” which we proposed to call CREST, and those with skin distal to elbows and knees but proximal to MCP or MTP joints, which we proposed to call CREAT (A instead of S for acrosclerosis) [13]. In that report, CREST patients had more anticentromere antibodies than those with CREAT and the presence of anti-Scl-70 occurred more frequently in the patients with CREAT than those with CREST. Small numbers did not allow for detection of clinical differences.

Over the past decades, many of the larger series [4–16] and particularly the EUSTAR database [17], one of the largest registries, have not differentiated subsets within the limited form (scleroderma sine scleroderma excluded).

Very recently however, in a very large series from a single center followed for years, the Hopkins group emphasized just this [18], showing differences between the two “limited” groups and stressing the higher prevalence of lung disease and anti-Scl-70 antibodies in the type 2 (Barnett) or intermediate form when compared with type 1, a behavior more similar to the diffuse form, and similar to what we had supported and Barnett proposed years ago. Their conclusion was that dividing into limited and diffuse as we do now left out an intermediate form with different clinical and serological characteristics [18].

Since over the years it was our feeling that this subsetting was clinically useful, we always registered extension of skin involvement in our patients, particularly identifying those with intermediate form within the lSSC. We therefore decided to examine the clinical and serologic characteristics of our patients with limited disease seen over the last decade to see if we could find differences within them according to the degree of skin involvement.

## 2. Patients and Methods

Cases were identified from one or more of the following sources.

Rheumatology Section database, patients with ICD 9 Code 710.1 (scleroderma) in the inpatient hospital database, patients with the item: scleroderma, CREST, or other synonyms as registered in the hospital's electronic clinical history; and laboratory database: patients with positive anticentromere, antinucleolar, or anti-Scl-70 results.

All medical records so identified were then manually reviewed to confirm diagnosis and obtain demographic data such as date of onset, clinical characteristics, and serologic profile.

Only cases fulfilling ACR 1980 criteria were included and were classified as diffuse or limited according to LeRoy's criteria. This difference in distribution was established considering the involvement of skin at its maximum extension at any point in the disease course. Within the limited subset, they were separated into sclerodactyly (only fingers) and acrosclerosis (fingers and up to elbows and/or knees, face) (Barnett's Types 1 and 2).

### 2.1. Definition of Organ Involvement

SSc clinical manifestations were considered to be present if predefined criteria were met during the course of the illness. Organ involvement definitions were the following: (1) upper gastrointestinal tract (one or more of the following: manometry with esophageal dysmotility, esophagram with gastroesophagic reflux or peristaltic alterations, or upper endoscopy with esophagitis, (2) pulmonary hypertension (PH): echocardiogram with estimated pulmonary systolic artery pressure equal to or greater than 40 mmHg or right heart catheterization with mean pulmonary artery pressure at rest over 25 mmHg, (3) interstitial lung disease (ILD): pulmonary interstitial disease observed in high resolution computerized tomography (HRCT) or pulmonary function tests with vital forced capacity (VFC) lower than 70% of the expected and/or carbon monoxide lung diffusion (DLCO) test under 80% of the expected, (4) echocardiographic alterations (aside from PH): left or right ventricular diastolic dysfunction in absence of arterial hypertension or pulmonary hypertension, respectively, or pericardial effusion, (5) digital ulcers: active digital ulcers or pitting scars confirmed by a physician, (6) renal involvement: history of scleroderma renal crisis (abrupt onset of accelerated arterial hypertension and/or rapidly progressive renal failure).

### 2.2. Autoantibodies

Laboratory detection methods were indirect immunofluorescence on HEp-2 cells (antinuclear antibodies, antinucleolar, and anticentromere). ANA dilutions greater than or equal to 1/80 were considered positive. ELISA was used for anti-topoisomerase I (anti-Scl-70). We are not able to detect anti-RNA polymerase antibodies in our institution.

### 2.3. Statistical Analysis

Incomplete data was analysed as missing data. Chi-square analysis was used to determine significant differences between sets of categorical data and Fisher's exact test when appropriate. *P* value <0.05 was considered statistically significant. Kaplan-Meier survival curve was used for survival analysis. Statistical analyses were performed using STATA version 10.

### 2.4. Ethical Approval

Our IRB does not require approval of retrospective analysis of codified data.

## 3. Results

Two hundred and thirty four SSc patients (216 females) fulfilled the established criteria. Female/male ratio was 12 : 1; 24% had diffuse SSc and 76% limited (64% sclerodactyly and 12% acrosclerosis). Total follow-up was 688 patient-years. Over half (55.1%) are still under our care and 17 died in our institution during this period. Ten-year survival rate was 80% for limited and 70% for diffuse variants, respectively (HR: 0.88, 95% CI: 0.7–1.1).

Table 1 shows clinical and serological profile of this cohort. Anti-Scl-70 was present in 16%, anticentromere in 53%, and nucleolar ANA in 7% of overall patients.

Further details of the whole cohort are published elsewhere [19].

Within the limited group, several characteristics in the acrosclerosis (Type 2) group were more similar to the diffuse patients than those with Type 1 (sclerodactyly). In patients with acrosclerosis, duration of Raynaud's was shorter, and they had significantly more anti-Scl-70 and less anticentromere antibodies than those with Type 1. In particular, interstitial lung disease (ILD) was significantly more prevalent in Type 2 group and similar to Type 3. Other characteristics did not reach statistical differences. Comparisons between patients with acrosclerosis and diffuse disease are presented in Table 2, showing no differences in clinical manifestations.

As described elsewhere, autoantibodies were associated with visceral involvement and also with clinical subset. In univariate analysis, anticentromere antibodies were associated with pulmonary hypertension, OR of 8.25 (CI 1.9–35.7), and inversely correlated with ILD (OR 0.18, CI 0.11–0.29). They were also associated with limited disease, OR 34.4 (CI 10.2–116.6). When performing multivariate analysis adjusting by disease subset, association between anticentromere and pulmonary hypertension was lost. All of the patients with PH had lSSc (11 with sclerodactyly and 1 with acrosclerosis) except for one patient with diffuse disease with anticentromere antibodies. In summary, all patients with isolated pulmonary hypertension had either limited cutaneous disease and/or anticentromere antibody.

In multivariate analysis, both anti-Scl-70 (OR 5.2, CI 1.5–17.6, *P* = 0.08) and diffuse disease (OR 4.1, CI 1.2–13.5, *P* = 0.021) were associated with ILD.

These findings are very similar to those recently described by Cottrell et al. within the “limited” patients, “dissecting” between what could be Barnett's type 1 and 2 subsets.

## 4. Discussion

Our results support the initial description by Barnett et al. that there is an “intermediate” form of SSc between the “fingers only” subset and the diffuse form. Some of these patients, albeit with limited scleroderma because skin involvement does not exceed elbows and/or knees, have a clinical behavior which can be described as intermediate between the strictly limited to fingers patients and those with diffuse disease. In particular, a sort of gradient from milder to more severe disease was confirmed in our patients, as suggested by Barnett many years ago. This was also shown by the Hopkins group in a much larger number of patients [18].

Indeed, in Type 2 as defined by Barnett, serology may be more similar to the diffuse type and different from Type 1. The “gradient” is also reflected by the accompanying antibodies which further define visceral involvement such as pulmonary hypertension and interstitial lung disease. These results appear to confirm that extension of skin involvement within limited SSc may identify two different subsets with clinical and serologic characteristics.

There is no doubt that early diagnosis of SSc is a necessity. Autoantibodies, in many cases present years prior to diagnosis and established skin disease, play an essential role here as predictors of clinical subsets and visceral involvement.

However, some of these are not widely available. Furthermore, many patients present with established disease, although early, and in these a clinical approach contemplating extent of skin involvement coupled with the classic autoantibodies may indeed be useful and support a division within the limited form which may have useful clinical implications.

A retrospective review of data in the large registries trying to document skin extension may or may not confirm this. The problem may arise in the fact that since the extension of skin involvement within limited disease was not considered important, this may not have been adequately registered.

## 5. Conclusions

Our smaller series completely agrees with the data shown by the Hopkins group with a very large number of patients. We believe identifying these differences within limited SSc is useful and “resuscitating” the intermediate form or Barnett type 2 within limited SSc may contribute to a better clinical assessment of systemic sclerosis.

## Figures and Tables

**Table 1:** Clinical and serological profiles of the 234 patients.

Subset of SSc	Limited (*n* = 178)		*P* (sclero. versus acro.)	Diffuse (*n* = 56)	*P* (limited versus diffuse)
	Sclerodactyly (*n* = 149)	Acrosclerosis (*n* = 29)			
Females, *n* (%)	142 (95,3)	25 (86,2)	0,06	49 (87,5)	0,13
Age at diagnosis, years, mean (SD)	59,8 (15,2)	54,9 (16,2)	0,14	53 (18,3)	**0,038**
Duration of Raynaud's prior to diagnosis, years, mean (SD)	9,2 (3,4)	5,5 (1)	**0,018**	1,9 (0,8)	**0,008**
Anti-Scl-70, *n* (%)	9 (6,4)	11 (39,3)	**<0,001**	18 (43,9)	**<0,001**
Anticentromere, *n* (%)	116 (82,3)	5 (17,9)	**<0,001**	2 (4,8)	**<0,001**
Nucleolar ANA, *n* (%)	8 (5,7)	1 (3,6)	0,54	9 (20,9)	**0,001**
Upper GI involvement, %	65,9	81,3	0,22	64,3	0,45
Interstitial lung disease, %	17,1	50	**<0,001**	65,3	**<0,001**
Pulmonary hypertension + interstitial lung disease, %	5,5	0	0,34	15,4	**0,03**
Isolated pulmonary hypertension, %	9,1	4,8	0,45	2,6	0,39
Cardiac ultrasound abnormalities (aside from PHT), %	5,7	4,5	0,65	10	0,26
Digital ulcers, %	26,9	34,5	0,44	32,1	0,86
Renal crisis, *n*	0	0		1	
Follow-up, patient/years-years (SD)	462 (3,1)	81,2 (3,1)		144,8 (3,6)	
Being followed presently, *n* (%)	88 (59,5)	15 (51,7)		26 (46,4)	
Deaths during follow-up, at our institution, *n*	11	2		4	

**Table 2:** Clinical and serological profiles comparing acrosclerosis and diffuse subsets.

Subset of SSc	Acrosclerosis (*n* = 29)	Diffuse (*n* = 56)	*P* (acro. versus diffuse)
Females, *n* (%)	25 (86,2)	49 (87,5)	1
Age at diagnosis, years, mean (SD)	54,9 (16,2)	53 (18,3)	0,7
Duration of Raynaud's prior to diagnosis, years, mean (SD)	5,5 (1)	1,9 (0,8)	0,2
Anti-Scl-70, *n* (%)	11 (39,3)	18 (43,9)	0,8
Anticentromere, *n* (%)	5 (17,9)	2 (4,8)	0,1
Nucleolar ANA, *n* (%)	1 (3,6)	9 (20,9)	0,07
Upper GI involvement, %	81,3	64,3	0,5
Interstitial lung disease, %	50	65,3	0,2
Pulmonary hypertension + interstitial lung disease, %	0	15,4	0,3
Isolated pulmonary hypertension, %	4,8	2,6	0,3
Cardiac ultrasound abnormalities (aside from PHT), %	4,5	10	0,6
Digital ulcers, %	34,5	32,1	1
Renal crisis, *n*	0	1	1
